# ASO Author Reflections: It’s Time to Take Dyspepsia Seriously

**DOI:** 10.1245/s10434-026-19880-y

**Published:** 2026-05-27

**Authors:** Kaitlyn J. Kelly

**Affiliations:** Department of Surgery, Division of Surgical Oncology, University of Wisconsin Hospital and Clinics, Madison, WI USA

## Past

Gastric cancer is the fifth most commonly diagnosed cancer worldwide and is a leading cause of cancer-related death globally, but is relatively low-incidence in the United States. High-incidence countries screen for gastric cancer with upper endoscopy (EGD) and routinely diagnose early-stage disease in patients.^1^ They have excellent outcomes and can even use minimally invasive techniques such as endoscopic submucosal dissection (ESD). Gastric cancer in low-incidence countries, such as the United States, tends to be advanced at presentation, and cure rates are low.^2^ This pattern has remained unchanged for decades, and most research efforts focus on more effective therapies for locally advanced and metastatic disease, but do not investigate what can be done to diagnose gastric cancer earlier in countries where the incidence does not justify screening. Recent consensus guidelines have broadened indications for diagnostic EGD to include new-onset dyspepsia after age 50 or 60 years, and to consider EGD screening for patients who are direct descendants from high-risk regions and those who have a family history of gastric cancer, even without a documented germline predisposition.^3^ With conflicting guidelines and ambiguous terminology such as “consider,” however, the utilization of diagnostic EGD for symptomatic patients remains highly variable and unstandardized.

## Present

This study comprehensively assessed a modern-day cohort of gastric cancer patients in the United States and demonstrated the highly variable use of diagnostic EGD^4^ despite the participation of the majority of the patients in colon cancer screening. The use of diagnostic EGD varied dramatically with presenting symptoms. Patients who presented with anemia or gastrointestinal bleeding received diagnostic EGD consistently and in a timely way, whereas patients who presented with dyspepsia, dysphagia, or reflux had variable use of EGD as the initial diagnostic study, and had a median wait time of 3 months to receive their first EGD.

**Fig. 1:**
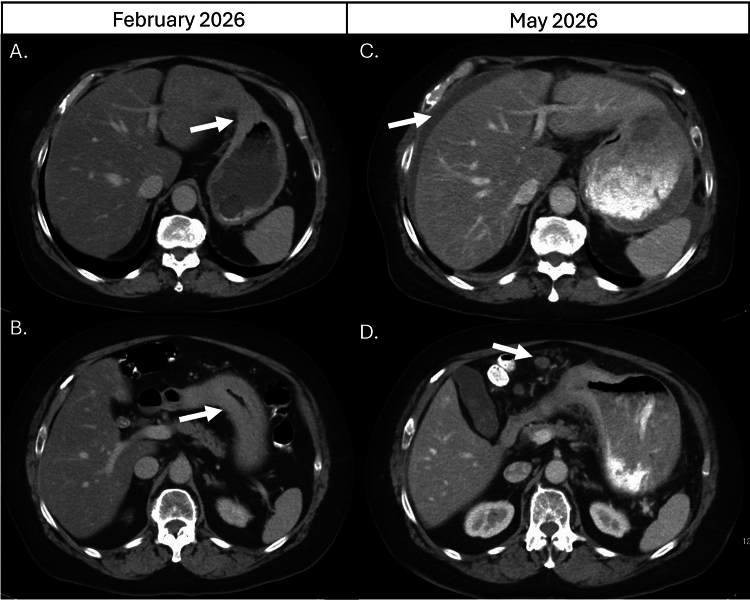
Images from a 68 year-old patient who presented with early satiety and belching (dyspepsia) and was first evaluated with a computed tomography in a rural setting. **A**, **B** A scan from February 2026 showed distal gastric wall-thickening without adenopathy or other findings. The same patient had persistent symptoms after proton pump inhibitor therapy, and a repeat scan exactly 3 months later in May 2026 demonstrated **C** interval development of malignant ascites and **D** bulky peri-gastric adenopathy

Figure 1 demonstrates computed tomography (CT) scans performed 3 months apart for a gastric cancer patient who was undergoing workup for dyspepsia at a community center. The images show rapid progression from an area of distal gastric wall-thickening in February of this year (Fig 1A, B) to the same wall-thickening with regional adenopathy and malignant ascites by May (Fig 1C, D), illustrating that 3 months is too long to wait for a patient with gastric cancer to receive a diagnosis. Finally, and not surprisingly, the study also showed that diagnosis by EGD compared with another method, such as cross-sectional imaging, was an independent predictor of improved overall survival regardless of disease stage. This highlights the importance of early tissue diagnosis and initiation of therapy for this biologically aggressive cancer.

## Future

Progress toward earlier diagnosis of gastric cancer and improved outcomes in low-incidence countries will require, at a minimum, standardized, clear indications for diagnostic EGD performed appropriately and in a timely manner not only for the indication of anemia and gastrointestinal (GI) bleeding, but also for dyspepsia, dysphagia, or reflux.^4^ In addition to improving timeliness and utilization of EGD for patients who present reporting symptoms, this could be taken a step further. A future study could focus on leveraging colon cancer screening as an opportunity to thoroughly question patients about upper GI symptoms that may be insidious or vague and that patients may not otherwise report themselves, with an endpoint of early-stage gastric cancer or other pathology that warrants intervention or surveillance. This could lead to selective diagnostic EGD in countries that screen for colon cancer but not gastric cancer, and could potentially save lives.
