# Correction: ASO Author Reflections: It’s Time to Take Dyspepsia Seriously

**DOI:** 10.1245/s10434-026-19945-y

**Published:** 2026-06-05

**Authors:** Kaitlyn J. Kelly

**Affiliations:** Department of Surgery, Division of Surgical Oncology, University of Wisconsin Hospital and Clinics, Madison, WI USA

**Correction to: Annals of Surgical Oncology** 10.1245/s10434-026-19880-y

In the original online version of this article, there were errors in the first sentence under the Future section. The correct sentence is as follows:

Progress toward earlier diagnosis of gastric cancer and improved outcomes in low-incidence countries will require, at a minimum, standardized, clear indications for diagnostic EGD performed appropriately and in a timely manner not only for the indication of anemia and gastrointestinal (GI) bleeding, but also for dyspepsia, dysphagia, and reflux.

The original article was corrected.

